# Coupling Between the Responses of Plants, Soil, and Microorganisms Following Grazing Exclusion in an Overgrazed Grassland

**DOI:** 10.3389/fpls.2021.640789

**Published:** 2021-07-26

**Authors:** Zhen Wang, Xiliang Li, Baoming Ji, Paul C. Struik, Ke Jin, Shiming Tang

**Affiliations:** ^1^National Agricultural Experimental Station for Soil Quality, Institute of Grassland Research, Chinese Academy of Agricultural Sciences, Ministry of Agriculture, Hohhot, China; ^2^The College of Forestry, Beijing Forestry University, Beijing, China; ^3^Centre for Crop Systems Analysis, Department of Plant Sciences, Wageningen University & Research, Wageningen, Netherlands; ^4^Department of Ecology, School of Ecology and Environment, Inner Mongolia University, Hohhot, China

**Keywords:** overgrazing, grazing exclusion, bacteria, fungi, community structure

## Abstract

Grazing exclusion is an effective management practice to restore grassland ecosystem functioning. However, little is known about the role of soil microbial communities in regulating grassland ecosystem functioning during long-term ecosystem restorations. We evaluated the recovery of a degraded semiarid grassland ecosystem in northern China by investigating plant and soil characteristics and the role of soil microbial communities in ecosystem functioning after 22 years of grazing exclusion. Grazing exclusion significantly increased the alpha diversity and changed the community structure of bacteria, but did not significantly affect the alpha diversity or community structure of fungi. The higher abundance of copiotrophic *Proteobacteria* and *Bacteroidetes* with grazing exclusion was due to the higher carbon and nutrient concentrations in the soil, whereas the high abundance of *Acidobacteria* in overgrazed soils was likely an adaptation to the poor environmental conditions. Bacteria of the *Sphingomonadaceae* family were associated with C cycling under grazing exclusion. Bacteria of the *Nitrospiraceae* family, and especially of the *Nitrospira* genus, played an important role in changes to the N cycle under long-term exclusion of grazing. Quantitative PCR further revealed that grazing exclusion significantly increased the abundance of nitrogen fixing bacteria (*nifH*), ammonia oxidizers (AOA and AOB), and denitrifying bacteria (*nirK* and *nosZ*1). Denitrifying enzyme activity (DEA) was positively correlated with abundance of denitrifying bacteria. The increase in DEA under grazing exclusion suggests that the dependence of DEA on the availability of NO_3_^–^ produced is due to the combined activity of ammonia oxidizers and denitrifiers. Our findings indicate that decades-long grazing exclusion can trigger changes in the soil bacterial diversity and composition, thus modulating the restoration of grassland ecosystem functions, carbon sequestration and soil fertility.

## Introduction

Livestock grazing is a common grassland management practice with far-ranging societal and environmental impacts. However, the effect of grazing on grassland ecosystem functioning primarily depends on the initial grazing intensity (Bardgett and Wardle, 2003). Overgrazing has been found to cause degradation of grassland ecosystem functioning and to reduce both plant productivity and soil fertility, resulting in nutrient depleted initial systems (Bardgett and Wardle, 2003; Chartier et al., 2013; Li et al., 2016; Yang et al., 2019). Grazing exclusion is an effective grassland management practice aimed at preventing grassland degradation and maintaining grassland ecosystem functions (Wang et al., 2018). Grazing exclusion can promote plant productivity (Deng et al., 2014), species diversity (Wu et al., 2014), soil fertility (Raiesi and Riahi, 2014), and soil microbial activity (Owen et al., 2015). Previous studies reported that approximately 20 years of grazing exclusion would be appropriate for restoring the degraded grasslands in northern China in terms of productivity and C and N storage (Qiu et al., 2013). Microbes are important contributors to the structure and functioning of ecosystems (Buyer et al., 2010); they drive nutrient transport and cycling in the soil (Wang Z. et al., 2019). However, there is not much literature reporting on the cumulative effects of long-term continuous overgrazing on the soil microbial community, and the role of the soil microbial community in the temporal progression of recovery from overgrazing remains unclear.

Grazing exclusion can have multiple effects on interactions among the soil microbiome, plant community and soil properties (Yang et al., 2018; Zhang X. et al., 2019). The plant community is an important driver during ecosystem restoration, affecting soil physicochemical properties by altering the input of litter, soil turnover of roots, and root exudation (Fry et al., 2016). In turn, the change in soil physicochemical properties influences the microbial communities (Liu et al., 2018). Microbes may, therefore, impact the growth of the plants in the sward because microbes can drive the transformation of organic substrates and the release of mineral elements during the process of ecosystem restoration (Wang Z. et al., 2019). However, we do not know the extent to which changes in the soil microbial community affect the impact of the grazing exclusion on plant growth and soil physicochemical properties.

On the other hand, soil microbial communities play an important role in biogeochemical processes, especially the N cycle. Microbes can support the N cycle via many of the critical processes, including nitrogen fixation, assimilation, nitrification and denitrification (Yang et al., 2013). Although grazing can strongly influence these N processes and related microbial groups (Patra et al., 2005; Xu et al., 2008; Xie et al., 2014), the effects of grazing on N cycling and microbial groups depend on its intensity (Bardgett and Wardle, 2003). Grazing exclusion eliminates the intake of livestock, which often leads to an increase in soil C and N storage, mainly due to the accumulation of plant litter on the soil surface (Wang Z. et al., 2019). The high soil N content under grazing exclusion increased soil ammonia availability, and substantially impacted the activity and communities of ammonia oxidizers (e.g., AOA, ammonia-oxidizing archaea; AOB, ammonia-oxidizing bacterial) (Lou et al., 2011). As a result of the change in nitrification [ammonium (NH_4_^+^) is converted to nitrite (NO_2_^–^) and then to nitrate (NO_3_^–^)], there is a change in the soil N cycle (Philippot et al., 2011).

Moreover, appropriate restoration (approximately 20 years of grazing exclusion) reduces soil compaction by avoiding animal trampling, which results in increased soil aeration and water-holding capacity (Kauffman et al., 2004; Blagodatsky and Smith, 2012). The denitrification activities of bacteria are suppressed in the presence of either NO_3_^–^ or NO_2_^–^ when animal trampling is avoided due to the changes in soil aeration (Hayatsu et al., 2008), which are linked to nitrite reductase and nitrous oxide reductase encoded by *nirK*, *nirS*, and *nosZ*1 (Pan et al., 2016). Therefore, further research is needed to investigate the mechanisms behind the influence of ecosystem rehabilitation on soil microbial community structure and function, especially related to the N cycle.

In this study, we investigate the long-term impact of grazing exclusion on the structure and functioning of soil microbial communities during ecosystem recovery. Considering the water-limited and oligotrophic environmental conditions in the semi-arid steppe (Pan et al., 2016; Wang Z. et al., 2019), the objectives of the current study were to analyze (1) which main environmental factors drive the shift of soil microbiome (bacterial or fungal community) during the recovery of a degraded ecosystem after the release of grazing pressure; and (2) whether the changes in the composition of soil microbial communities play a large role in the recovery of the biogeochemical function.

## Materials and Methods

### Study Area

**FIGURE 1:**
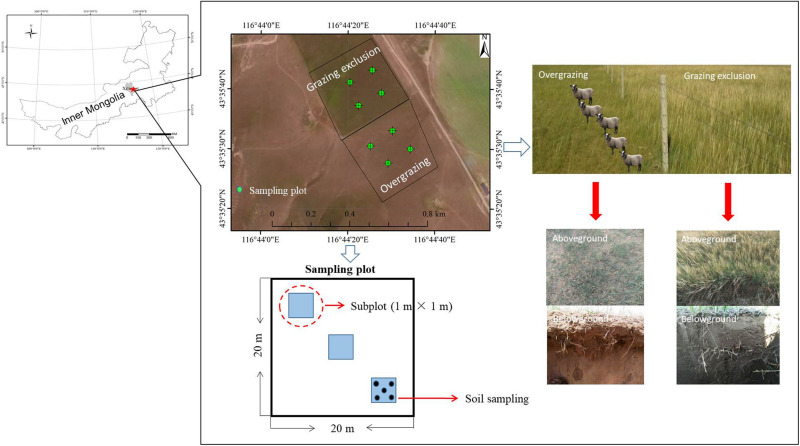
Effect of overgrazing and grazing exclusion on the semiarid steppe.

The field experiment was carried out at the Inner Mongolia Grassland Ecosystem Research Station (N 43°35′30″ to 43°35′42″, E 116°42′20″ to 116°42′35″, Figure 1), which represents the semiarid steppe ecosystem. The long-term mean annual precipitation (1953–2009) was 335 mm, with more than 70% of precipitation falling during the growing season (May−August). The mean annual temperature is 0.4°C, ranging from the lowest monthly average temperature of −21.4°C in January to the highest of 18.0°C in July. The soil is classified as Calcic Chernozems (IUSS Working Group WRB, 2006), with similar physiochemical properties of chestnuts and calcic chernozems in a previous study (Steffens et al., 2008). The basic soil properties of the study areas were found to comprise 17.3% clay, 34.8% silt and 47.9% sand by using the hydrometer method (Kettler et al., 2001), the soil organic carbon (SOC) was 21.10 g kg^–1^ assessed using dichromate oxidation (Nelson and Sommers, 1982) and soil total nitrogen (TN) content was 1.85 g kg^–1^ assessed using an automatic Kjeldahl instrument (Kjeltec 8400, FOSS Corporation, Denmark).

### Experimental Design and Sampling

To explore the role of grazing exclusion on grassland ecosystem function, we compared the soil community in grazed plots where grazing had been eliminated for 22 years. Our study was established as a pair of large-scale plots which involved pseudo-replication limited in space-for-time substitution. However, this challenge is surmountable as has been reported in previous ecological studies (Walker et al., 2010; Blois et al., 2013; Lü et al., 2015). Grazing and grazing exclusion plots had similar soil types, topographies, altitudes, slope gradients and slope aspects (Supplementary Table 1). The grazed plots were located adjacent to the grazing exclusion (or restoration) plots, and had been grazed year-round for more than 30 years. Grazing begins in early-June and ends in early-October. The stocking rate in the grazing plots was approximately 3 sheep ha^–1^ y^–1^, which was two times higher than the local stocking rate of 1.5 sheep ha^–1^ y^–1^ (Li et al., 2015). The dominant plant species in the grazing site were *Stipa grandis* (grass), *Artemisia frigida*, (forb), and *Cleistogenes polyphylla* (grass) (Supplementary Table 2 and Supplementary Figure 1), while the grazing exclusion site was dominated by *Leymus chinensis* (grass) and *Stipa grandis* (grass) after 22 years of restoration (Supplementary Figure 1). Bare soil increased by 24.25% under overgrazing in our study.

Four 20 m × 20 m plots were randomly established at each site, using a paired sampling method within the overgrazed and grazing exclusion treatments (Figure 1). The two sites were never fertilized or mowed during the management. The plots were randomly assigned within 200 m of each other. Three 1 m × 1 m subplots were established along a transect within each plot for investigation and sampling in mid-August 2018. In the middle of August during peak biomass, we measured vegetation ground coverage, aboveground net primary productivity (ANPP), plant height, and species richness (SR). All aboveground plant materials were harvested to the ground surface (including living aboveground biomass, standing litter, and ground litter) in the quadrat (1 m × 1 m). We separated plant aboveground tissue (living aboveground biomass) from standing litter of the previous year and litter on the ground. We used the Shannon−Wiener index (*H* = −ΣP_*i*_lnP_*i*_) and plant species richness to estimate the diversity of the plant communities, where P_*i*_ is the ratio of the coverage of each species to the coverage of all species. Harvested biomass was determined by drying the aboveground tissues at 65°C for 48 h (Wang et al., 2014). Aboveground net primary productivity (ANPP) was calculated as the sum of the aboveground biomass for all plant species (Wang et al., 2016). Soil bulk density (BD) was measured by using the USDA (1972) method. Five soil cores (3.5 cm in diameter) were extracted and segmented in depth increments of 0–5, 5–10, 10–15, and 15–20 cm. The core was composited at different depth increments, air-dried, then ground until passable through a 2-mm screen. Soil samples were collected from the top 20 cm of the soil profile as soil cores (3.5 cm in diameter). Five soil cores were collected from each subplot after removing aboveground biomass, and then a total of 15 soil cores (five each from three subplots) were combined to make one composite sample. We eliminated roots, stones, litter, and debris from each soil sample by using a 2-mm sieve, before field storage and transport to the laboratory on ice in a cooler. The composite soil samples were divided into three subsamples. The first subsample was air dried for physicochemical analysis. The second subsample was stored at 4°C to determine soil NH_4_^+^ and NO_3_^–^ concentrations and transported to the laboratory for immediate analysis, as well as for microbial C and N biomass determination. The third subsample was stored at −80°C for DNA extraction.

### Analysis of Soil Physicochemical Properties

Soil water content (SW) of each composite soil sample was measured by weighing before and after drying at 105°C for 24 h. Soil pH was determined by shaking a soil/water suspension (1:1 weight/vol, DI water) for 30 min (Fierer et al., 2006). Soil organic carbon (SOC) was measured using dichromate oxidation (Nelson and Sommers, 1982). The total nitrogen content (TN) was determined using an automatic Kjeldahl instrument (Kjeltec 8400, FOSS Corporation, Denmark). The NH_4_^+^ and NO_3_^–^ concentrations in the soil subsamples were determined by digestion with 2 mol L^–1^ KCl at a 1:3 ratio (w:v) and analyzed by a flow injection analyzer (FIAstar 5000, FOSS Analytical, Höganäs, Sweden). Soil available phosphorus (AP) was measured using the Kelowna method as described by Van Lierop (1988) using a solid to liquid ratio of 1:5. The soil total phosphorus concentration (TP) in the extracting solution was measured using an Astoria auto-analyzer (Clackamas, OR, United States).

### Soil Microbial Biomass and Enzymatic Activities

We used a fumigation extraction method to measure the soil microbial C and N biomass (Vance et al., 1987). We fumigated 25 g of the oven-dry equivalent of field-moist at 25.8°C for 24 h with CHCl_3_. The soil was added to 100 ml of 0.5 M potassium sulfate, shaken at 200 rpm for 1 h, and then filtered (0.2 μm) after removing the fumigant. An additional 25 g of non-fumigated soil was simultaneously extracted. The soil organic carbon (SOC) and soil total nitrogen (TN) contents of the extracts were measured using a Liqui TOCII analyzer (Elementar Analyses system, Hanau, Germany).

Urease activity was measured using a urea solution as the substrate and incubation at 37°C for 24 h (a spectrophotometer was employed to determine the NH_4_^+^-N concentration at 578 nm) (Nannipieri et al., 1980). Nitrate reductase activity was determined using KNO_3_ solution as the substrate and incubation at 25°C for 24 h (a spectrophotometer was employed to determine the NO_2_^–^ concentration at 520 nm) (Daniel and Curran, 1981).

The potential nitrification rate (PNR) was assessed according to the procedures described in Kurola et al. (2005). Twenty mL of phosphate buffered saline (PBS) solution was added as substrate to 5 g of fresh soil in a 50 mL centrifuge tube with 1 mmol L^–1^ (NH_4_)_2_SO_4_ (100 ppm N), and then the centrifuge tubes were placed at room temperature in the dark for 24 h. Eight grams NaCl, 0.2 g KCl, 0.2 g Na_2_HPO_4_ and 0.2 g NaH_2_PO_4_ were mixed in about 800 mL of water as PBS solution (pH = 7.1). To inhibit nitrite oxidation, potassium chlorate (at a final concentration of 10 mmol L^–1^) was then added to the centrifuge tubes. After incubation, 5 mL of 2 mol L^–1^ KCl was added to the tubes to extract NO_2_-N. After centrifugation, the sulfonamide and naphthalene oxalamide were used as reagents to analyze the optical density of the supernatant by the presence of NO_2_-N at 545 nm.

Soil denitrifying enzyme activity (DEA) was measured according to the method of Hart et al. (1994). A fresh soil sample (equivalent to 15 g dry soil) was added to a 250 ml plasma flask with a 100 mL solution of 1.5 mM (NH_4_)_2_SO_4_ (100 ppm N) and 1 mM phosphate buffer (pH = 7.2). The flask was incubated at room temperature with continuously stirring (180 rpm). Samples were extracted at 2, 4, 8, 12, and 24 h during incubation. The concentrations of NO_2_^–^ and NO_3_^–^ were measured in the samples by using a continuous flow analyzer. The DEA rate was calculated based on the slope of the regression of NO_2_^–^ plus NO_3_^–^ concentration against time.

### Soil DNA Extraction and Sequencing

Before sequencing the 16S rRNA and internal transcribed spacer (ITS) gene sequences, all soil composite samples (0.5 g) were processed for DNA extraction with the FAST DNA Spin Kit for Soil (MP Biomedicals, Santa Ana, CA, United States) according to the manufacturer’s instructions. Two separate DNA extractions from 0.5 g of soil were then merged together for polymerase chain reaction (PCR) amplification. The bacterial PCR primers were 515F (5′-GTGCCAGCMGCCGCGGTAA-3′) and 806R (5′-GGACTACHVGGGTWTCTAAT-3′) with the target 16S V4 region (Zheng et al., 2018). The fungal ITS1 region was amplified using the primers ITS5-1737F (5′-GGAAGTAAAAGTCGTAACAAGG-3′) and ITS2-2043R (5′-GCTGCGTTCTTCATCGATGC-3′) (Bellemain et al., 2010). Both sets of primers contained a 6-bp error-correcting barcode (8 – 4 for overgrazing and 4 for grazing exclusion) that was unique to each sample for the identification of individual samples in mixture Illumina HiSeq sequencing runs (Novogene Bioinformatics Technology Co., Ltd., Beijing, China). PCR amplicons were further purified with a DNA purification kit (BioFlux, Japan), and the concentrations were determined using spectrometry (NanoDrop-1000, United States). Amplicons from different samples were then mixed and purified with Qiagen Gel Extraction Kit (Qiagen, Germany) to achieve equal mass concentrations in the final mixture, and sent to Novogene Co., Ltd., Tianjing, China, for sequencing library construction and pair-end sequencing using the Illumina HiSeq sequencing system (Illumina, United States). All amplicon sequencing data have been deposited in the NCBI SRA under the accession number PRJNA695426 (bacteria) and PRJNA695427 (fungi). After sequencing, 250 bp paired-end reads were generated and assigned to samples based on their unique barcode sequence, followed by cutting off the barcode and primer sequence. Paired-end reads were merged using FLASH (V1.2.7) (Magoč and Salzberg, 2011). Quality filtering (Bokulich et al., 2013) was performed to obtain only the high-quality clean tags according to the QIIME quality control process (V1.7.0) (Caporaso et al., 2010). Chimera sequences were removed by comparing with the reference database (Gold database) using the UCHIME algorithm (Edgar, 2013). Sequences with ≥97% similarity were assigned to the same operational taxonomic unit (OTU). The SILVA (bacteria) and UNITE (fungi) databases were used to assign taxonomic information to each OTU representative sequence. OTU abundance information was normalized using a standard sequence number corresponding to the sample with the least number of sequences (44,254 for bacteria and 37,223 for fungi) and used for subsequent analysis of alpha diversity and beta diversity (Supplementary Figure 2).

### Real-Time Quantitative PCR

We quantified the amount of the target sequence in genomic DNA by using real-time quantitative PCR. After the quality control, nitrogen-fixing genes were quantified using different primers. The primer pairs and thermal-cycling conditions of real-time quantitative PCR are described in detail in Supplementary Table 3. The total bacterial community was quantified using the 16S rRNA gene (34lF/534R). The total fungal community was quantified using the ITS gene (ITS4/ITS5). The abundances of nitrogen-fixing (*nifH*), nitrification (AOA and AOB), and denitrification genes (*nirK*, *nirS*, and *nosZ*1) were obtained for subsequent comparative analysis. The amplification of PCR products was monitored by measuring specific fluorescence signals using the dsDNA-specific fluorescent dye SYBR Green I (measured after the extension phase). The inhibition tests were performed when we ran the qPCR assay. We conducted an inhibition test to determine whether samples were amplified with the same efficiency as the standard. In the qPCR inhibition test, each sample to be tested was spiked with a standard. The Ct value of the spiked sample was then compared with the Ct value of the pure standard. The percent inhibition (or actual % efficiency) was calculated according to the following formula: 1 − [(Ct sample – Ct standard)/Ct standard] × 100. In our study, a calculated inhibition of 1–2% was observed in some samples and was accepted without dilution. All quantitative PCR reactions were performed in triplicate with an ABI 7900 system. We added Bovine Serum Albumin (BSA) (10 mg/mL) to these PCR reaction mixes to reduce the inhibitory effects of co-extracted polyphenolic soil compounds. Briefly, 10 μL of reaction mixes contained 5 μL Power qPCR PreMix (GENEray, GK8020) and primers, 1 μL BSA, 1 μL 20×-diluted DNA template (1.2–5.0 ng) and 3 μL Milli-Q water. We analyzed the products from quantitative PCR reactions, and only accepted one specific peak of each target sequence in the dissociation curves. A standard curve of DNA copies was created using the concentration on the *X*-axis (in copies/μL of 10-folded dilution series) and CT value on the *Y*-axis (Greilhuber et al., 2005). Every dot represents a CT value from duplication of standard DNA. We performed a linear regression and obtained the logarithm equation from each standard curve. The equation Eff = [10^(–1/slope)^ – 1] was used to calculate the amplification efficiencies, which resulted in the following values: bacterial 16S rRNA 90%, fungal ITS 91%, *nifH* 92%, AOA-*amoA* 93%, AOB-*amoA* 85%, *nirK* 87%, *nirS* 98%, and *nosZ*1 99%.

### Statistical Analysis

Prior to statistical analysis, plant characteristic data in the three 1 m × 1 m subplots were averaged. All of the statistical analyses were conducted using R software (Version 3.2.4) (R Core Team, 2016). Univariate analysis of variance (ANOVA) was used to examine the effects of grazing exclusion on plant characteristics (ANPP and diversity), soil physicochemical metrics (SOC, TN, TP, NH_4_^+^, NO_3_^–^, and AP), soil enzymatic activities (urease, nitrate reductase, potential nitrification rate, and denitrifying enzyme activity), soil microbial characteristics (C and N biomass, and diversity), bacterial 16S rRNA, abundance, fungal ITS abundance, and N cycle functional genes (*nifH*, AOA-*amoA*, AOB-*amoA*, *nirK*, *nirS*, and *nosZ*1). A suite of alpha diversity indices, including number of OTUs, Chao1, Shannon–Wiener, Simpson, ACE, and good-coverage, were calculated for analyzing species diversity with QIIME, and visualized with R software. Significance tests were based on Tukey’s honestly significant difference (HSD) between any two compared objects. Statistical significance was defined as *P*-values in the Tukey’s HSD corrected with the Benjamini-Hochberg false discovery rate.

Additionally, PERMANOVA was used to examine the effects of grazing exclusion on soil bacterial and fungal community compositions based on weighted UniFrac distances. The weighted UniFrac distances were employed to assess whether two communities were different using the QIIME software (Version 1.7.0). A principal coordinate analysis (PCoA) was used to assess the differences in the structures of microbial communities among different grazing treatments based on weighted UniFrac metric matrices using the VEGAN package (Oksanen et al., 2013) in R software (R Core Team, 2016). The relative abundances of different taxa in the bacterial and fungal community compositions between grazing and grazing exclusion were also determined by PERMANOVA using the VEGAN package in R software. The effects of grazing exclusion on the statistical difference between the relative abundance of bacterial and fungal taxa were analyzed using STAMP software. Significance tests were based on unpaired Student’s *t*-tests to identify differences between any two compared objects.

Pearson’s correlation analyses were conducted to identify the environmental factors accounting for the patterns of microbial alpha diversity (number of OTUs, Chao1, Shannon–Wiener, Simpson, ACE, and good-coverage) and the gene abundances associated with N fixation (*nifH*), nitrification (AOA*-amoA* and AOB*-amoA*) and denitrification (*nirK*, *nirS*, and *nosZ*). Heat maps were generated to show the relationships between the relative abundances of different taxa in soil microbial community compositions (bacterial/fungal) and environmental variables (plant characteristics and soil chemical properties) and microbial C and N biomass. The heat maps were generated in R.3.2.4 using the pheatmap package and the correlation analysis was carried out using the psych package of R.3.2.4. A multivariate regression trees (MRT) analysis was carried out to identify the most important biotic and abiotic factors for bacterial and fungal community composition using the mvpart package (De’Ath, 2002). Stepwise multiple linear analyses were used to examine the relationships between the gene abundances associated with N fixation (*nifH*), nitrification (AOA*-amoA* and AOB*-amoA*), denitrification (*nirK*, *nirS* and *nosZ*1), and soil properties (SW, pH, NH_4_^+^, NO_3_^–^, SOC, and TN).

## Results

### The Effect of Grazing Exclusion on Plant, Soil, and Microbial Activity

**FIGURE 2:**
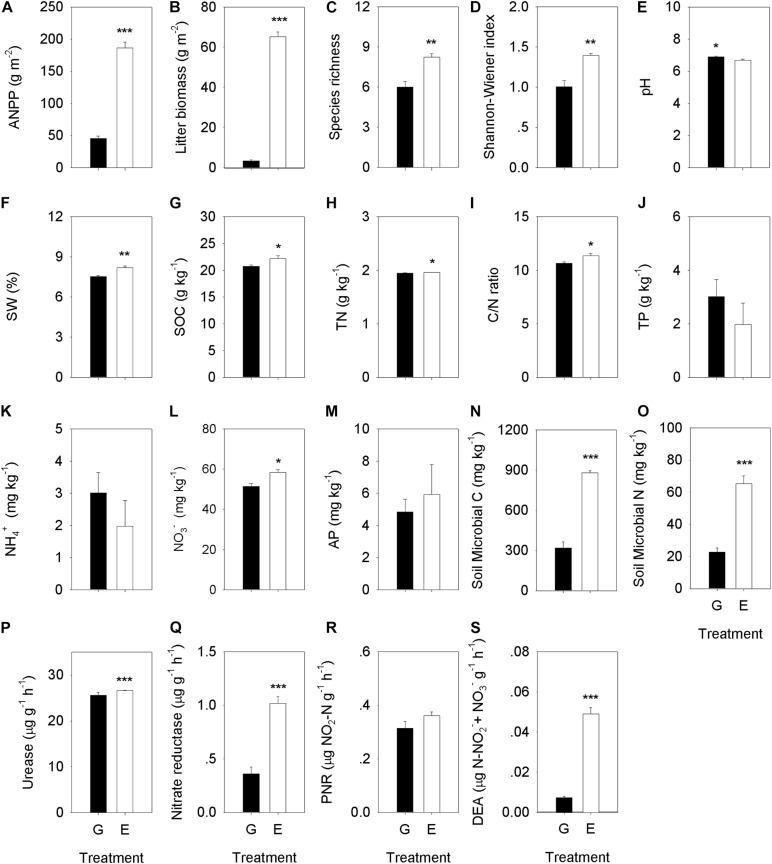
Comparison analysis of the plant community characteristics, soil properties, soil microbial C and N, and soil enzyme activities between the overgrazing and grazing exclusion treatments. Plant community characteristics include **(A)** ANPP, **(B)** litter biomass, **(C)** species richness, and **(D)** Shannon–Wiener index. Soil properties include **(E)** soil pH value, **(F)** soil water content (SW), **(G)** soil organic carbon content (SOC), **(H)** soil total nitrogen content (TN), **(I)** soil C/N ratio, **(J)** soil total phosphorus content (TP), **(K)** soil NH_4_^+^ content, **(L)** soil NO_3_^–^ content, and **(M)** soil available phosphorus content (AP). Soil microbial biomass include **(N)** microbial C, and **(O)** microbial N. Soil enzyme activities include: **(P)** urease (UR), **(Q)** Nitrate reductase (NR), **(R)** the potential nitrification rate (PNR), and **(S)** soil denitrifying enzyme activity (DEA). G, overgrazing; E, grazing exclusion. Values represent the mean ± standard error (*n* = 4). Significance levels are indicated as: *P < 0.05, **P < 0.01, and ***P < 0.001.

Grazing exclusion significantly changed plant community composition and increased plant species biomass (*P* < 0.05), including *Leymus chinensis* (*P* < 0.001), *Stipa grandis* (*P* < 0.001), *Achnatherum sibiricum* (*P* = 0.006), and *Agropyron cristatum* (*P* < 0.001) (Supplementary Figure 1), ANPP (*P* < 0.001), species richness (*P* = 0.003) and the Shannon−Wiener index (*P* = 0.003) (Figure 2 and Supplementary Table 4). Moreover, there was a significantly positive effect of long-term grazing exclusion to soil physicochemical characteristics (Figure 2). Grazing exclusion significantly increased SW (*P* = 0.007), SOC (*P* = 0.043), TN (*P* = 0.006), soil C/N ratio (*P* = 0.048), NO_3_^–^ (*P* = 0.014), nitrate reductase activity (*P* = 0.003), and DEA (*P* < 0.001) (Figure 2 and Supplementary Table 4). Grazing exclusion significantly reduced soil BD at different depth increments, including 0–5 (*P* = 0.007), 5–10 (*P* < 0.001), 10–15 (*P* = 0.035), and 15–20 cm (*P* = 0.041) (Supplementary Figure 3).

### Effect of Grazing Exclusion on Microbial Biomass and Composition

Grazing exclusion significantly increased soil microbial biomass C (*P* < 0.001) and N (*P* < 0.001) (Figure 2 and Supplementary Table 4). Bacterial alpha diversity was higher in grazing exclusion soils than in overgrazed ones, including higher OTU richness (*P* = 0.035), and higher values for the *H′* (*P* = 0.039), Chao1 (*P* = 0.039), and ACE diversity (*P* = 0.029) (Table 1). However, there were no significant differences in the fungal alpha diversity index between the overgrazing and the grazing exclusion soils (Table 1).

**TABLE 1:** The comparison of the alpha diversity of bacteria and fungi between overgrazing and grazing exclusion.

	Bacteria			Fungi		
	Overgrazing	Grazing exclusion	*P*-value	Overgrazing	Grazing exclusion	*P*-value
OTU richness	**3990.00 ± 25.16**	**4193.25 ± 69.07**	**0.0348**	1302.00 ± 78.21	1219.25 ± 114.87	0.5733
*H*′	**9.77 ± 0.07**	**10.00 ± 0.06**	**0.0385**	7.84 ± 0.38	7.12 ± 0.59	0.3402
Simpson	0.99 ± 0.01	0.99 ± 0.01	0.5370	0.98 ± 0.01	0.94 ± 0.02	0.1681
Chao1	**5027.81 ± 184.58**	**5649.88 ± 107.16**	**0.0269**	1752.88 ± 128.81	1627.63 ± 114.34	0.4345
ACE	**5224.60 ± 110.94**	**5661.46 ± 105.73**	**0.0292**	1795.70 ± 114.77	1719.43 ± 127.36	0.6722
Goods_coverage	0.97 ± 0.01	0.96 ± 0.01	0.0528	0.97 ± 0.01	0.97 ± 0.01	0.7329

*Results reported as the mean ± standard error (n = 4). P < 0.05 values in bold indicates significant differences between grazing and grazing exclusion. H′, Shannon–Wiener diversity.*

**FIGURE 3:**
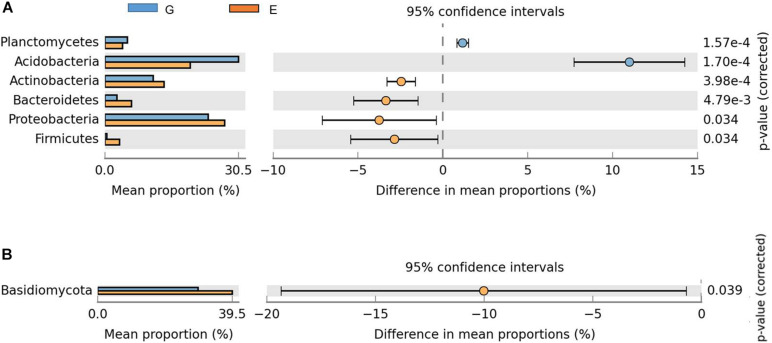
Comparison of phyla with significant differences between the overgrazing and grazing exclusion treatments. The data were visualized using STAMP (error bars represent Welch’s t-interval). **(A)** Bacteria, **(B)** fungi. Bars on the left represent the proportion of each phylum abundance (bacterial and fungal) in the treatments. Bacterial abundance differences with a *q*-value of <0.05 were considered to be significant. *Q*-values were determined using the Benjamini-Hochberg adjustment for *p*-values.

The change of relative abundances in bacterial groups revealed shifts in dominant taxa between overgrazing and grazing exclusion (*P* < 0.05, Figure 3). The dominant bacterial phyla in the overgrazing and grazing exclusion included *Acidobacteria* (29.13% vs. 18.54%), *Proteobacteria* (22.53% vs. 25.97%), *Verrucomicrobia* (10.57% vs. 12.21%), *Actinobacteria* (10.59% vs. 12.86%), *Gemmatimonadetes* (10.33% vs. 8.51%), *Bacteroidetes* (2.63% vs. 5.80%), *Planctomycetes* (4.95% vs. 3.81%), and *Firmicutes* (4.49% vs. 3.17%; Figure 3A). *Acidobacteria* (*P* < 0.001) and *Planctomycetes* (*P* < 0.001) showed greater relative abundance in the overgrazed soils, while *Proteobacteria* (*P* = 0.034), *Actinobacteria* (*P* < 0.001), *Bacteroidetes* (*P* = 0.005), and *Firmicutes* (*P* = 0.034) showed greater abundance in grazing exclusion soils (*P* < 0.05; Figure 3A). Grazing exclusion significantly increased the relative abundance of *Betaproteobacteria* (*P* = 0.001) and *Deltaproteobacteria* (*P* = 0.015) (Supplementary Figure 4). Compared with overgrazing, grazing exclusion significantly increased the relative abundance of some families, including *Gaiellaceae*, *Solirubrobacteraceae*, *Nocardioidaceae*, and *Conexibacteraceae* (all belonging to the phylum *Actinobacteria*), *Sphingomonadaceae*, *Rhodobiaceae, Polyangiaceae*, *Sinobacteraceae*, and *Haliangiaceae* (all belonging to the phylum *Proteobacteria*), and *Nitrospiraceae* (phylum *Nitrospirae*), but significantly decreased the relative abundance of *mb2424* (phylum *Acidobacteria*) (*P* < 0.05; Supplementary Figure 5). Grazing exclusion significantly increased the abundance of some genera, such as *Mycobacterium* (phylum *Actinobacteria*), *Afifella*, *Sphingomonas*, and *Lysobacter* (all belonging to the phylum *Proteobacteria*), and *Nitrospira* (phylum *Nitrospirae*) (*P* < 0.05; Supplementary Figure 6).

**FIGURE 4:**
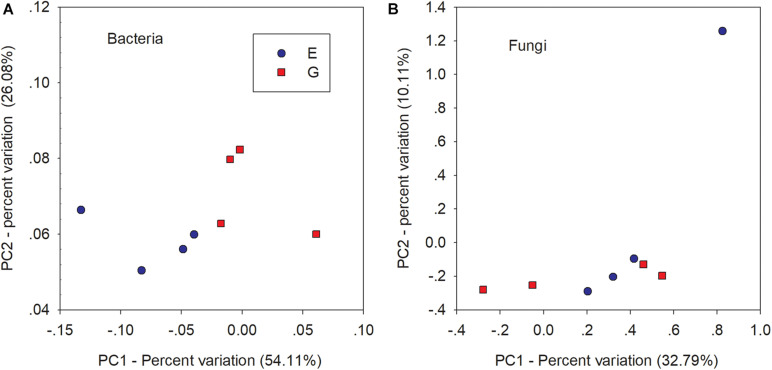
Principal Coordinates Analysis (PCoA) of bacteria **(A)** and fungi **(B)** dissimilarity based on weighted UniFrac distance matrix between overgrazing (red squares) and grazing exclusion (blue circles).

Compared with overgrazing, grazing exclusion significantly increased the abundance of *Basidiomycota* (*P* = 0.039; Figure 3B). Grazing exclusion significantly increased the relative abundance of some families, including *Lasiosphaeriaceae* and *Herpotrichiellaceae* (phylum *Ascomycota*), as well as *Auriscalpiaceae* (phylum *Basidiomycota*) (*P* < 0.05; Supplementary Figure 7). The PCoA ordination revealed differences in bacterial communities, which showed a clear separation between overgrazing and grazing exclusion along the first PCoA 1 axis (*P* < 0.05, Figure 4A). No difference was found for the fungal community (Figure 4B).

### Associations of Bacterial and Fungal Diversity With Soil and Plant Properties

**FIGURE 5:**
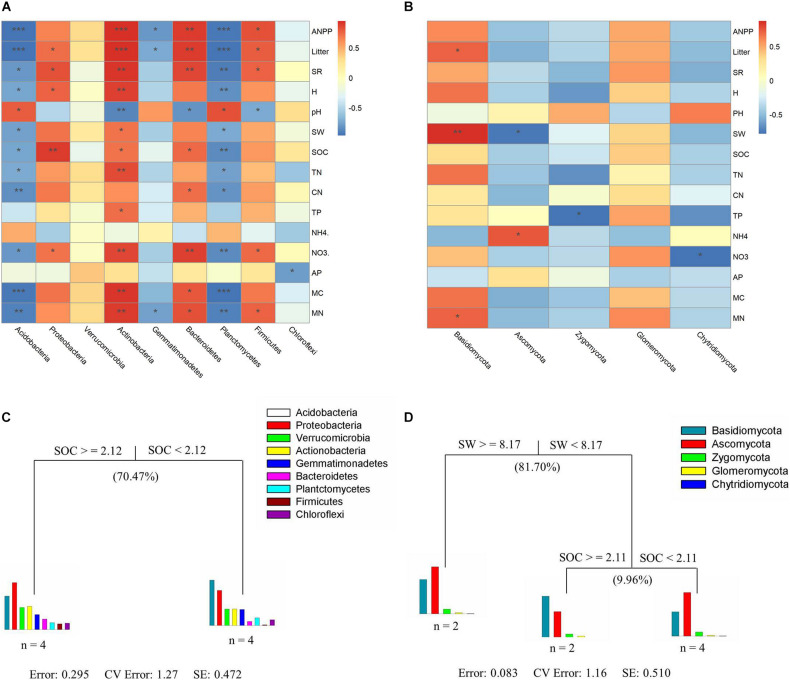
Correlations between biotic and abiotic factors and dominant bacterial and fungal phyla **(A,B)**. Multivariate regression tree analysis of environmental factors on the patterns of soil bacterial **(C)** and fungal **(D)** community composition. The number of soil samples included in the analysis is shown under the bar plots. Plant community characteristics include the ANPP, litter (litter biomass), SR (species richness), and *H* (Shannon–Wiener index). Soil properties include the pH (soil pH value), soil water content (SW), SOC (soil organic carbon content), TN (soil total nitrogen content), CN (soil C/N ratio), TP (soil total phosphorus content), NH4 (soil NH_4_^+^ content), NO3 (soil NO_3_^–^ content), and AP (soil available phosphorus content). Soil microbial variables include the MC (microbial C) and MN (microbial N). Significance levels in heat maps analysis are indicated as: *P < 0.05, **P < 0.01, and ***P < 0.001.

In this study, plant characteristics (SR, ANPP, and litter biomass) and soil properties (SOC, C/N ratio, and NO_3_^–^) were positively related to the alpha diversity indices of the soil bacterial community (the number of OTUs, *H*′, Chao1 and ACE) (*P* < 0.05, Supplementary Table 5). For bacterial communities, significant correlations based on heat map analyses were found between soil or plant characteristics and bacterial taxa (except *Chloroflexi*) (Figure 5A). For fungal communities, no significant relationship was found between environmental factors (except SW) and fungal taxa (Figure 5B). MRT analysis was used to explain the relative effects of plant and soil properties on the bacterial and fungal community composition from all samples (Figures 5C,D). A visual tree in the MRT analysis showed two splits in the bacterial community based on plant and soil properties (Figure 5C; cross-validated relative error 1.27 and 0.742, respectively), whereas fungal community composition showed three splits in a visual tree (Figure 5D; cross-validated relative error 1.16 and 0.510, respectively). SOC was the major factor affecting soil bacterial community composition and explained 70.47% of the variation (Figure 5C). SW and SOC together explained 91.66% of the variation in fungal community composition (Figure 5D), and we found that SW (which explained 81.70%) was the key factor affecting changes in the fungal community composition.

### Effect of Grazing Exclusion on the Abundances of Microbial Groups

**FIGURE 6:**
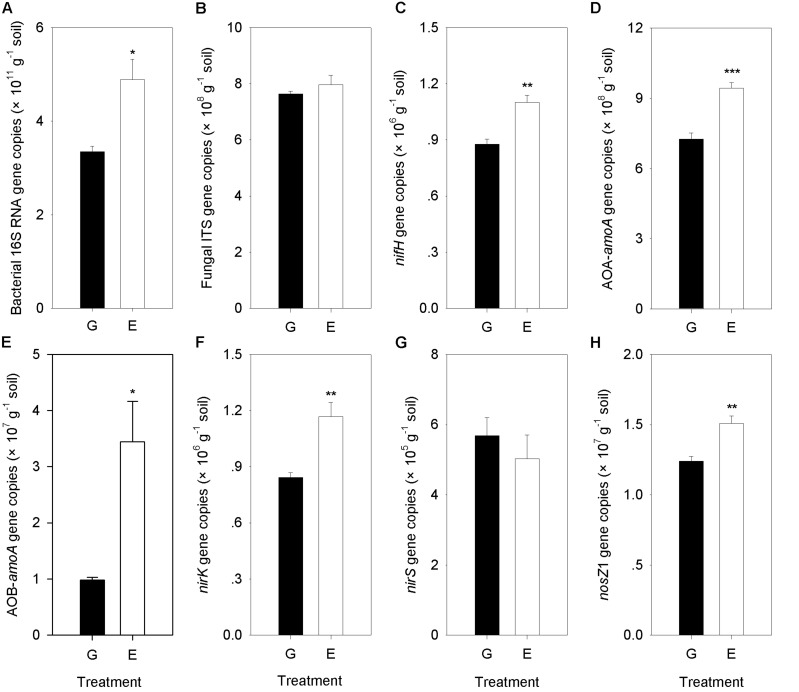
Comparison of Bacterial 16S RNA gene, Fungal ITS gene and the N cycling gene abundances (*nifH*, AOA-*amoA*, AOB-*amoA*, *nirK*, *nirS*, and *nosZ*1) between overgrazing and grazing exclusion. G, overgrazing; E, grazing exclusion. Values represent the mean ± standard error (*n* = 4). Significance levels are indicated as: *P < 0.05, **P < 0.01, and ***P < 0.001. Bacterial 16S RNA gene copies **(A)**, Fungal ITS gene copies **(B)**, nifH gene copies **(C)**, AOA-amoA gene copies **(D)**, AOB-amoA gene copies **(E)**, nirK gene copies **(F)**, nirS gene copies **(G)**, and nosZ1 gene copies **(H)**.

**FIGURE 7:**
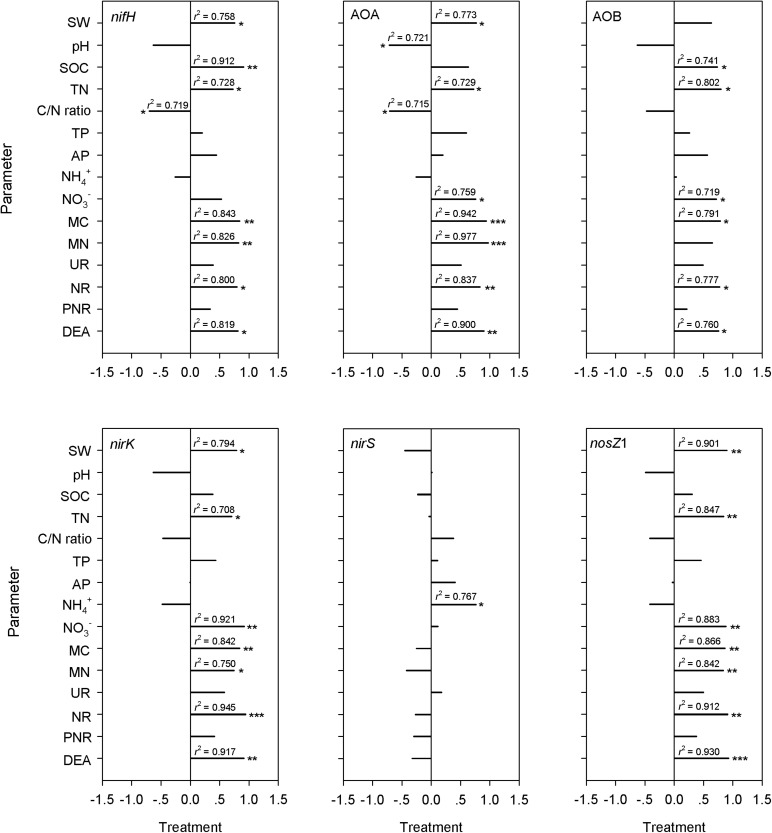
Pearson correlation coefficients between N cycling gene abundances (*nifH*, AOA-*amoA*, AOB-*amoA*, *nirK*, *nirS*, and *nosZ*1) and biotic and abiotic factors. The correlations were derived for SW, water/moisture content of soil samples; pH, soil pH value; SOC, soil organic content of the soil samples; TN, total nitrogen concentration of soil samples; C/N ratio, (soil C/N ratio); TP, total phosphorus content of soil samples; AP, available phosphorus content of soil; NH_4_^+^, soil NH_4_^+^ content; NO_3_^–^, soil NO_3_^–^ content; MC, soil microbial C; MN, soil microbial N; UN, urease; NR, nitrate reductase; PNR, the potential nitrification rate; DEA, soil denitrifying enzyme activity. Significance levels are indicated as: *P < 0.05, **P < 0.01, and ***P < 0.001.

A significant difference between overgrazed and grazing exclusion soils was found for bacterial 16S rRNA gene copy numbers and six key functional N gene families (nitrification, denitrification and N fixation) (*P* < 0.05; Figure 6). The grazing exclusion significantly increased the bacterial 16S rRNA gene copy numbers and *nifH* gene abundance (*P* = 0.003; Figure 6). For nitrification genes, the abundances of AOA-*amoA* (*P* < 0.001) and AOB-*amoA* (*P* = 0.014) both increased in the grazing exclusion soils (Figure 6). For denitrification genes, the grazing exclusion significantly increased the abundance of the *nirK* (*P* = 0.007) and *nosZ*1 (*P* = 0.008) genes (Figure 6). Soil physicochemical characteristics (SOC, TN and NO_3_^–^) increased linearly with the gene abundances of the N cycle (*nifH*, AOA, AOB, *nirK*, and *nosZ*) (*P* < 0.05, Figure 7). Soil microbial biomass (C and N) showed a linear and positive correlation with gene abundances of the N cycle (*nifH*, AOA, AOB, *nirK*, and *nosZ*) (*P* < 0.05, Figure 7). The soil enzyme activities (NR and DEA) also indicated a positive, linear relationship with the N cycle gene abundances (*nifH*, AOA, AOB, *nirK*, and *nosZ*) (*P* < 0.05, Figure 7). Stepwise multiple regression analyses showed that the abundance or relative abundance of six key functional N gene families could be explained by the four soil physicochemical factors, namely, SW, SOC, soil TN content, and soil NO_3_^–^ content (Table 2). SW and SOC together accounted for 98% of the spatial variation in the *nifH* gene (*P* < 0.001; Table 2). SW explained 53% of the spatial variation in the abundance of AOA genes (*P* = 0.025; Table 2). TN alone explained 58% of the abundance of AOB genes (*P* = 0.017; Table 2). Soil NO_3_^–^ content alone explained 83% of the spatial variation in the gene abundance of *nirK* (*P* < 0.001), whereas NO_3_^–^ alone explained 52% of the spatial variation in the gene abundance of *nirS* (*P* = 0.026; Table 2). Both SW and TN content were responsible for 92% of the spatial variation in the abundance of *nosZ*1 (*P* < 0.001; Table 2).

**TABLE 2:** Stepwise multiple regression analysis of the relationships between independent variables and gene abundance of *nifH*, AOA, AOB, *nirK*, *nirS*, and *nosZ*1.

Functional genes	Results	*R*^2^	*F*	*P*
*nifH*	*y* = 0.717 × (SOC) + 0.438 × (SW) – 2.07	0.98	177.27	<0.001
AOA	*y* = 0.773 × (SW) – 1.01	0.53	8.88	0.025
AOB	*y* = 0.802 × (TN) – 3.28	0.58	10.84	0.017
*nirK*	*y* = 0.921 × (NO_3_^–^) – 1.23	0.83	33.69	<0.001
*nirS*	*y* = 0.061 × (NO_3_^–^) + 0.38	0.52	8.59	0.026
*nosZ*1	*y* = 0.556 × (SW) + 0.498 × (TN) – 6.88	0.92	39.58	<0.001

*SW, soil water content; SOC, soil organic carbon content; TN, soil total nitrogen content; NO_3_^–^, soil NO_3_^–^ content.*

## Discussion

### Grazing Exclusion Altered Bacterial Diversity

Our results revealed that bacterial alpha diversity (i.e., OTU richness, *H′*, Chao1, and ACE) significantly increased in response to grazing exclusion (Table 1), which was consistent with previous findings that both the *H′* and ACE indices of soil bacterial diversity significantly increased with recovery age (Wu et al., 2014; Zhang et al., 2018). The observed positive relationship between soil nutrient content (i.e., SOC and NO_3_^–^) and bacterial alpha diversity in our study supported previous findings (Cheng et al., 2016; Wang et al., 2018). Additionally, bacterial alpha diversity was not directly associated with plant diversity in a previous study (Millard and Singh, 2010), but in our study plant diversity had a positive relationship with bacterial alpha diversity (Supplementary Table 5). Our results suggested that the management practice of grazing exclusion (appropriate restoration) increased bacterial alpha diversity and are consistent with a recent study on semiarid grasslands (Zhang et al., 2018), in which bacterial alpha diversity was higher after 25 years of grazing exclusion than in other sites (0, 10, and 35 years of grazing exclusion). We attributed this to the fact that the nutrients required by soil bacteria are usually obtained from plant litter, release of root exudates and root decay of live plants (Tang et al., 2020). High plant species diversity increased plant community production, which was related with greater litter accumulation on the soil surface and enhanced C inputs to soil (Buyer et al., 2010). Our results further showed that the effect of plant diversity on soil nutrient concentration also impacted soil bacterial diversity among soil bacteria taxa (Figures 3, 5). High plant diversity may contribute to greater diversity of plant-derived resources (El Moujahid et al., 2017), and provide more opportunities for soil microbes to specialize in different resources (Kinkel et al., 2011). Thus, changes in bacterial alpha diversity were closely associated with plant diversity (i.e., species richness and Shannon–Wiener index). The relationship between the diversities of plant species (above-ground) and soil bacteria (below-ground) is a key point of the ecosystem biodiversity (Yang et al., 2020).

### Grazing Exclusion Changed Bacterial Community Composition

With regard to the effects of grazing exclusion on the microbial community composition, we observed that different microbial taxa exhibited different behaviors. A previous study showed that intensive grazing increased the relative abundances of *Proteobacteria*, *Bacteroidetes* and *Firmicutes* (Patra et al., 2005; Xun et al., 2018; Zhang et al., 2020b). However, in our study, the relative abundances of *Actinobacteria*, *Proteobacteria*, *Firmicutes*, and *Bacteroidetes* increased under grazing exclusion by increasing soil carbon. These induced changes of grazing exclusion in bacterial taxa are consistent with previous studies (Cheng et al., 2016; Wang et al., 2018).

There are some possible explanations for the changes in bacterial community diversity and composition due to grazing exclusion. First, direct effects of avoiding animal trampling under grazing exclusion on soil carbon have been associated with increased soil air permeability (Wang Z. et al., 2019). Indirect carbon storage induced under grazing exclusion via plant litter accumulation has been demonstrated in the Loess Plateau (Cui et al., 2019). The increased abundances of bacterial taxa are for copiotrophic groups under grazing exclusion, which are generally fast-growing and positively linked to SOC concentration (Leff et al., 2015; Zhang et al., 2018). Thus, the increase in SOC after 22 years of grazing exclusion resulted in a shift in the bacterial community from oligotrophic groups to copiotrophic groups, characterized by decreases in *Acidobacteria* phyla abundances and increased abundances in the *Actinobacteria* phylum and the *Bacteroidetes* phylum, and the *Betaproteobacteria* and *Deltaproteobacteria* class (Figure 4; Supplementary Figure 3).

Although a previous study showed that grazing increased the relative abundance of both the *Firmicutes* and *Bacteroidetes* phyla through livestock dung, such results were observed under moderate grazing (Zhang et al., 2020b). Compared to grazing exclusion and moderate grazing, the limited amount of herbage under overgrazing may cause livestock to consume more energy while foraging and this might result in reduced quantities of palatable, high-quality and highly productive grasses, such as *Leymus chinensis*, *Stipa grandis*, and *Melissilus ruthenicus* (L.) Peschkova (Supplementary Figure 1 and Supplementary Table 1). Overgrazing decelerated nutrient cycling by the dominance of nutrient-poor or chemically defensive species (e.g., *Salsola collina* and *Tribulus terrestris*) with low litter quality (Bai et al., 2012). Under nutrient-deficient conditions, low-quality litter decreased nutrient concentration and root biomass, which often affects the amount of C-rich substrates exuded into the rhizosphere (McNaughton, 1985). Thus, microbial activity and the use of stored nutrients were inhibited under overgrazing. As a result, overgrazing accelerated the loss of soil nutrients, and consequently reduced SOC concentration. Both the *Firmicutes* and *Bacteroidetes* phyla consisted of copiotrophic bacteria (Leff et al., 2015), which are fast growing and positively correlated with SOC concentration, thus, explaining the reductions in the relative abundance of *Bacteroidetes* and *Firmicutes* under overgrazing.

Additionally, the heat map analyses also showed that the change in the relative abundances of main bacterial phyla was related to plant characteristics, soil properties and soil microbial biomass (Figure 5). For example, the *Actinobacteria* phylum can promote plant growth by making nutrients/substrates (e.g., phosphorus and nitrogen) available to host plants and producing various plant hormones to prevent plant infections (Liu et al., 2017). The *Proteobacteria* phylum can accumulate soil N content to promote plant growth because many N-fixing bacteria belong to the *Proteobacteria* phylum (Spain et al., 2009). The negative correlation between the abundance of the *Acidobacteria* phylum and other parameters (plant characteristics, soil properties, and soil microbial biomass) is due to the fact that the *Acidobacteria* phylum contains microbes that usually grow rapidly in a nutritionally poor environment (Koyama et al., 2014). Moreover, the increased abundance of the *Proteobacteria* family (*Sphingomonadaceae*, *Sinobacteraceae*, *Haliangiaceae*, *Polyangiaceae*, and *Rhodobiaceae*), the *Actinobacteria* family (*Gaiellaceae*, *Solirubrobacteraceae*, *Streptomycetaceae*, and *Conexibacteraceae*), and the decreased abundance of the *Acidobacteria* family (*mb2424*) under grazing exclusion, also led to the change in bacterial community composition (Supplementary Table 4).

### Lack of Fungal Response Under Grazing Exclusion

Unlike diversity and composition of the bacterial community, fungal community composition did not significantly differ between overgrazing and grazing exclusion (Patra et al., 2005), suggesting that the bacterial community may develop faster than the fungal community (Figure 5). Our results are consistent with a previous study conducted by Brown and Jumpponen (2015), who found that the fungal community did not respond to succession age, while the bacterial community strongly responded, as determined by a phylogenetic diversity analysis. Bacteria have a more diverse physiology than fungi, thus they successfully colonize during the grassland ecosystem restoration (Zhang et al., 2018). Compared to bacteria, fungi are more dependent on C and N sources. Fungi may not have many available niches before accumulating enough organic matter in the succession process (Prewitt et al., 2014). Additionally, MRT analysis showed that SW was a key factor affecting the change in fungal community composition based on the MRT analysis. Our results are in accordance with recent studies (Tedersoo et al., 2014), in which water availability affected plant community productivity, and subsequently impacted the quantity and quality of the input of plant residues supporting the soil fungal community. Grazing exclusion enhanced the relative abundance of the *Basidiomycota* phyla (saprotroph) (Figure 3B), which was likely due to the relatively higher SW, MN, litter biomass, SOC, and plant biomass under grazing exclusion (Yang et al., 2018).

### Bacterial Response Under Grazing Exclusion

Removal of grazing elicited changes in soil microbial community structure that led to improved biogeochemical functions and higher soil fertility. The change in bacterial phyla may be due to increased soil C and N substrates by litter accumulation (Zeng et al., 2017), which is in agreement with the higher litter biomass, OC, and TN contents detected in our study (Figure 2). The increase in the relative abundance of the family *Sphingomonadaceae* may improve the oxygen availability and may change the soil physical environment (e.g., decreased soil bulk diversity) by avoiding animal trampling (Wang Z. et al., 2019). As a result, grazing exclusion increased the SOC, which is associated with greater litter input into soil. The MRT analysis also identified SOC as the predominant factor driving the change in the composition of the soil bacterial community. Therefore, changes in the composition of specific microbial groups likely played an important role in the recovery of the biogeochemical functions as it is supported by the strengthened relationship between microbial phylogenetic composition and soil fertility since the release from the exclusion of grazing.

The changes in the abundance of N cycle functional genes provided a glimpse of the functional potentials of microbial communities under grazing exclusion. We observed a dramatic increase in *nifH* gene abundance with the grassland ecosystem recovery, which was related to plant and soil properties. Previous studies showed that *nifH* genes primarily from aerobic and facultatively anaerobic organisms, which belong to three bacterial phyla (*Proteobacteria*, *Firmicutes*, and *Actinobacteria*) (Gaby and Buckley, 2014). The higher relative abundance of *Proteobacteria*, *Firmicutes*, and *Actinobacteria* led to an increase in the abundance of *nifH* genes due to the increased soil fertility under grazing exclusion (Meyer et al., 2013). Additionally, our results are consistent with those of a previous study (Poly et al., 2001), in which the increase in the soil C/N ratio drove N fixation under grazing exclusion. Moreover, potential acidity is related to pH, a well-established factor affecting the diversity of microbial communities (Jesus et al., 2009), which also increased *nifH* gene abundance under grazing exclusion.

For the nitrifier communities, the abundance of AOA was much greater than that of AOB in our study. Our results agree with those of a recent study, in which AOA played a major role in the nitrification of acidic soils (Zhang et al., 2012). Additionally, AOA rather than AOB is favored in the low-fertility and low-nitrogen environments in this semiarid grassland (Figure 2), which is in line with observations in other ecosystems (Shrewsbury et al., 2016; Assémien et al., 2017). Grazing exclusion increased the abundance of nitrification genes (AOA and AOB) in soils, reflecting a response to remove the grazing trampling. Nitrification genes in grazing exclusion grassland soils increased (Figure 6), which might be attributed to the removal of grazing trampling that promotes the oxygen-requiring nitrification process (Pan et al., 2016). In our study, SW was correlated with changes in the abundance of the AOA gene (Table 2). This agreed with a previous study in the Inner Mongolia Steppe (Xie et al., 2014; Ding et al., 2015), in which AOA gene abundance rapidly responded to the water content. The recovery of the soil NO_3_^–^ content is tightly related to changes in the gene abundance of AOB (Table 2), which was associated with the abundance of *Nitrospiraceae* (Supplementary Figure 5). Our results agree with the findings of Wang J. et al. (2019), who reported that the abundance of AOB was correlated with *Nitrospira* abundance. Effectively, grazing exclusion increased the abundance of nitrification genes.

Interestingly, grazing exclusion did not change PNR but increased the abundance of the AOA and AOB communities, suggesting the PNR was not necessarily associated with the abundance of ammonia-oxidizers in our study (Yin et al., 2019). Le Roux et al. (2013) showed that the correlations between the abundance of ammonia oxidizers (AOA and AOB) and PNR were weak in grasslands. Our results are consistent with previous studies (Nicol et al., 2008; Yin et al., 2019), in which the activities of ammonia-oxidizers were related with enzyme function rather than with the abundance of functional genes.

For the denitrifier communities, the gene abundances (*nirK* and *nosZ*1) showed positive relationships with DEA, which was associated with the general enhancement of substrates (e.g., NO_3_^–^). Our results are in line with a previous study, which indicated that DEA can predict the change in denitrifier (*nirK*) abundance (Morales et al., 2010; Attard et al., 2011; Zhang X. et al., 2019). The higher DEA in our grazing exclusion soils suggested that ammonia oxidizers (higher AOA and AOB abundances under grazing exclusion) provided substrates (e.g., NO_3_^–^) to denitrifiers (*nirK* and *nosZ*1), and DEA relies on the availability of NO_3_^–^ production.

The increased abundance of *nirK* observed under grazing exclusion supports the findings of previous studies, in which the nitrate reducer communities increased during the ecological recovery of the grassland (Song et al., 2019). Consistent with this interpretation (Ding et al., 2015), the SW, soil nutrients (e.g., NO_3_^–^) and oxygen were the most important factors mediating the gene abundances of denitrifiers. Grazing exclusion significantly increased the abundance of the *nirK* gene, but no changes were observed in the abundance of the *nirS* gene (Figure 6), which was inconsistent with the findings of a previous study in a semiarid steppe (Pan et al., 2016). Our results are consistent with the observations of a recent study in Tibetan alpine meadows (Xie et al., 2014), in which the different responses of the abundances of *nirK*- and *nirS*-nitrite reducers to grazing intensity were attributed to niche differentiation between these two groups of denitrifiers for different ecosystems (Assémien et al., 2019). Additionally, nitrate reductase activity was determined by *nirK*, and there was a positive relationship between enzyme activities and the changes in gene abundances of denitrifier genes (Barrena et al., 2017).

## Conclusion

Grazing exclusion in the semiarid steppe caused significant changes in soil properties, bacterial diversity and community structure, but there were no significant alterations in fungal diversity and community structure. The diversity and structure of the bacterial community indicated a positive linear relationship with plant and soil functioning during restoration of these grassland ecosystems. Our results clearly demonstrated a positive relationship between the abundances of denitrifying functional genes (*nirK* and *nosZ*1) and DEA during restoration of grassland ecosystems. Our results suggest that grazing exclusion can initiate changes in the soil bacterial community that facilitate the recovery of ecosystem functions in grasslands.

## Data Availability Statement

All amplicon sequencing data have been deposited in the NCBI SRA under the accession numbers SRR13612594-SRR13612601 (bacteria) and SRR13612480-SRR13612487 (fungi).

## Author Contributions

ZW, KJ, BJ, XL, and ST conceived and designed the research. ZW, XL, KJ, and ST conducted the experiment. ZW, BJ and PS analyzed and interpreted the data. ZW, KJ, BJ, and ST wrote the manuscript. All authors discussed and approved the final version of the manuscript.

## Conflict of Interest

The authors declare that the research was conducted in the absence of any commercial or financial relationships that could be construed as a potential conflict of interest.

## Publisher’s Note

All claims expressed in this article are solely those of the authors and do not necessarily represent those of their affiliated organizations, or those of the publisher, the editors and the reviewers. Any product that may be evaluated in this article, or claim that may be made by its manufacturer, is not guaranteed or endorsed by the publisher.
