# Supplementary material for: Coupling Between the Responses of Plants, Soil, and Microorganisms Following Grazing Exclusion in an Overgrazed Grassland
Source: Front Plant Sci. 2021 Jul 26;12:640789. doi: 10.3389/fpls.2021.640789 (PMC8351616; doi:10.3389/fpls.2021.640789)
Supplement: Supplementary file 1 [file Data_Sheet_1.docx]

**Supplemental materials**

**TABLE S1** Geographical characteristics of the study sites. The soil of both sites is a Calcic Chernozem.

| Site | Altitude (m) | Slope gradient | Slope aspect |
| --- | --- | --- | --- |
| Overgrazing | 1207 | 18 | E20’N |
| Grazing exclusion | 1219 | 18 | E21’N |

**TABLE S2** Species components of plant functional groups at the experimental site in the desert steppe of the Xisu Banner, Inner Mongolia.

| Plant functional groups | Species |
| --- | --- |
| Grass | *Stipa klemezii* Roshev., *Carex duriuscula* C.A.Mey. |
|  | *Cleistogenes songorica* (Roshev.) Ohwi, *Eragrostis pilosa* (L.) Beauv. |
| Forb | *Artemisia frigida* Willd.*, Allium polyrhizum* Turcz. ex Regel., *Convolvulus ammannii* Desr., *Heteropappus altaicus* (Willd.) Novopokr., *Corispermum hyssopifolium* L., *Salsola collina* Pall*.*, *Tribulus terrestris* L., *Bassia dasyphylla* (Fisch. & C. A. Mey.) Kuntze |

**TABLE S3** Primer sequences used in quantitative real time PCR.

| **Target Primer set gene** | **Primer name** | **Primer sequence (5 sed)** | **Product size (bp)** | **Time, voltage and temperature** | **Reference** |
| --- | --- | --- | --- | --- | --- |
| 16S rRNA | 34l-F | CCTACGGGAGGCAGCAG | 194 | 95 °C /15 min, 1 cycle of 95 °C / 15 s, 60 °C / 30 s, 72 °C / 30 s, 75 °C / 30 s, 35 cycles | Smits et al., 2004 |
|  | 534-R | TTACCGCGGCTGCTGGCAC | | |  |
| Fungus ITS | ITS 4 | TCCTCCGCTTATTGATATGC | 206 | 96 °C / 3 min, 35 cycle of 94 °C / 30 s, 55°C / 30 s, 72 °C / 1 min, 72 °C / 7 min | He et al., 2015 |
|  | ITS 5 | GGAAGTAAAAGTCGTAACAAGG | | | |
| *nifH* | nifH-F | AAAGGYGGWATCGGYAARTCCACCAC | 458 | 97 °C / 4 min, 15 cycles of 96 °C / 20 s, 72 °C / 40 s, 25 cycles of 94 °C/ 20 s, 58 °C / 35 s | Rosch et al., 2002 |
|  | nifH-R | TTGTTSGCSGCRTACATSGCCATCAT | | | |
| *AOB-amoA* | *amoA*-1F | GGGGTTTCTACTGGTGGT | 530 | 95 °C /10 min, 1 cycle of 95 °C /30 s, 57° / 30 s, 72 °C /40 s, 82 °C / 10 s, 45 cycles | Rotthauwe et al., 1997 |
|  | *amoA*-1R | CCCCTCKGSAAAGCCTTCTTC | | |  |
| *AOA-amoA* | *CrenamoA*F | ATGGTCTGGCTWAGACG | 625 | 95 °C / 10 min, 1 cycle of 95 °C / 30 s, 53 °C / 60 s, 72 °C / 60 s, 50 cycles | Tourna et al., 2008 |
|  | *CrenamoA*R | GCCATCCATCTGTATGTCCA | | |  |
| *nirK* | *nirK*-876F | GGMATGGTKCCSTGGCA | 164 | 95 °C /15 min, 1 cycle of 9 5°C /15 s, 62 °C / 30 s, 72 °C / 30 s, 80 °C / 30 s, 6 cycles, 95 °C / 15 s, 58 °C / 30 s, 72 °C / 30 s, 8 0°C / 30 s, 35 cycles | Henry et al., 2004 |
|  | *nirK*-1040R | GCCTCGATCAGRTTRTGG | | |  |
| *nirS* | *nirS*-4QF | GTSAACGYSAAGGARACSGG | 413 | 95 °C /15 min, 1 cycle of 95 °C /15 s, 62 °C / 30 s, 72 °C / 30 s, 80 °C / 30 s, 6 cycles, 95 °C / 15 s, 58 °C / 30 s, 72 °C / 30 s, 80 °C / 30 s, 35 cycles | Kandeler et al., 2006 |
|  | *nirS*-6QR | GASTTCGGRTGSGTCTTSAYGAA | | |  |
| *nosZ* | *nosZ*-1840F | CGCRACGGCAASAAGGTSMSSGT | 264 | 95 °C /15 min, 1 cycle 95 °C /15 s, 64 °C / 30 s, 72 °C / 30 s, 80 °C / 30 s, 6 cycles, 95 °C/15 s, 60 °C / 30 s, 72°C / 30 s, 80 °C / 30 s, 35 cycles | Henry et al., 2006 |
|  | *nosZ*-2090R | CAKRTGCAKSGCRTGGCAGAA | | |  |

**References**

He, S., Niu, Q., Li, Y., Nie, Y., Hou, M. (2017) Factors associated with the diversification of the microbial communities within different natural and artificial saline environments. *Ecological Engineering* 83: 476-484.

Henry, S., Baudoin, E., López-Gutiérrez, J.C., Martin-Laurent, F., Brauman, A., Philippot, L. (2004) Quantification of denitrifying bacteria in soils by *nirK* gene targeted real-time PCR. *J Microbiol Meth* 59: 327–335.

Henry, S., Bru, D., Stres, B., Hallet, S., Philippot, L. (2006). Quantitative detection of the nosZ gene, encoding nitrous reductase, and comparison of the abundances of 16S rRNA, narG, nirK, and nosZ genes in soils. *Applied and Environmental Microbiology* 72: 5181e5189.

Kandeler, E., Deiglmayr, K., Tscherko, D., Bru, D., Philippot, L. (2006). Abundance of narG, nirK, and nosZ genes of denitrifying bacteria during primary successions of a glacier foreland. *Applied and Environmental Microbiology* 72, 5957e5962.

Rosch, C., Mergel, A., Bothe, H. (2002). Biodiversity of denitrifying and dinitrogen-fixing bacteria in an acid forest soil. *Applied and Environmental Microbiology* 68: 3818-3829.

Rotthauwe, J., Witzel, K. & Liesack, W. (1997). The ammonia monooxygenase structural gene amoA as a functional marker: molecular fine-scale analysis of natural ammonia-oxidizing populations. .*Applied and Environmental Microbiology* 63: 4704- 4712.

Smits, T.H.M., Devenoges, C., Szynalski, K., Maillard, J., Holliger, C. (2004). Development of a real-time PCR method for quantification of the three genera *Dehalobacter*, *Dehalococcoides*, and *Desulfitobacterium* in microbial communities. *Journal of Microbiological Methods* 57: 369e378.

Tourna, M., Freitag, T.E., Nicol, G.W., Prosser, J.I. (2008). Growth, activity and temperature responses of ammonia-oxidizing archaea and bacteria in soil microcosms. *Environmental Microbiology* 10: 1357e1364.

**TABLE S4** Comparitive analysis of the plant community characteristics, soil properties, soil microbial C and N, and soil enzyme activities between the overgrazing and grazing exclusion treatments.

|  | Grazing |  |  | Grazing exclusion | | *P* value |
| --- | --- | --- | --- | --- | --- | --- |
|  | Mean | SE |  | Mean | SE |  |
| **Plant community characteristics** | |  |  |  |  |  |
| ANPP (g m^-2^) | **45.558** | **3.718** |  | **186.285** | **9.048** | **<.0001** |
| Little biomass (g m^-2^) | **3.458** | **0.432** |  | **65.140** | **2.396** | **<.0001** |
| SR | **6.000** | **0.408** |  | **8.250** | **0.250** | **0.003** |
| plant *H*' | **1.006** | **0.077** |  | **1.396** | **0.024** | **0.003** |
| **Soil chemistry properties** |  |  |  |  |  |  |
| pH | **6.890** | **0.045** |  | **6.695** | **0.051** | **0.028** |
| SW (%) | **7.533** | **0.084** |  | **8.188** | **0.140** | **0.007** |
| SOC (g kg^-1^) | **2.074** | **0.027** |  | **2.219** | **0.049** | **0.043** |
| TN (g kg^-1^) | **0.195** | **0.000** |  | **0.196** | **0.000** | **0.006** |
| C/N ratio | **11.340** | **0.256** |  | **10.639** | **0.145** | **0.049** |
| TP (g kg^-1^) | 0.055 | 0.008 |  | 0.067 | 0.002 | 0.223 |
| NH_4_^+^ (mg kg^-1^) | 3.010 | 0.642 |  | 1.979 | 0.798 | 0.354 |
| NO_3_^-^ (mg kg^-1^) | **51.413** | **1.602** |  | **58.401** | **1.257** | **0.014** |
| AP (mg kg^-1^) | 4.838 | 0.791 |  | 5.913 | 1.861 | 0.614 |
| **Microbial biomass** |  |  |  |  |  |  |
| Microbial C (mg kg^-1^) | **319.144** | **43.531** |  | **879.476** | **16.259** | **<.0001** |
| Microbial N (mg kg^-1^) | **22.618** | **2.668** |  | **65.159** | **4.929** | **<.0001** |
| **Soil enzyme activities** |  |  |  |  |  |  |
| UR (μg g^-1^ h^-1^) | 25.613 | 0.658 |  | 26.609 | 0.145 | 0.190 |
| NR (μg g^-1^ h^-1^) | **0.359** | **0.059** |  | **1.015** | **0.064** | **<.0001** |
| PNR (μg NO_2_-N g^-1^ h^-1^) | 0.315 | 0.030 |  | 0.361 | 0.015 | 0.215 |
| DEA (μg N-NO_2_^-^+NO_3_^-^ g^-1^ h^-1^) | **0.007** | **0.001** |  | **0.049** | **0.003** | **<.0001** |

Plant community characteristics include ANPP, litter biomass, species richness (SR), and Shannon-Wiener index (plant *H*'). Soil properties include soil pH value, soil water content (SW), soil organic carbon content (SOC), soil total nitrogen content (TN), soil C/N ratio, soil total phosphorus content (TP), soil NH_4_^+^ content, soil NO_3_^-^ content, and soil available phosphorus content (AP). Soil microbial biomass includes microbial C and microbial N. Soil enzyme activities include urease (UR), nitrate reductase (NR), the potential nitrification rate (PNR), and soil denitrifying enzyme activity (DEA).

Values represent the mean ± standard error (n = 4).

**TABLE S5** Pearson’s correlation coefficients for plant characteristics (SR, Shannon-Wiener, and ANPP), soil (SW, pH, OC, TN, TP, NH_4_^+^, NO_3_^-^, and AP), soil microbial C (MC) and N (MN) biomass, and bacteria and fungi diversity (OTUs, *H’*, Chao1, and ACE).

| Community | Parameters | OTUs |  |  | *H*' |  |  | Chao1 |  |  | ACE |  |
| --- | --- | --- | --- | --- | --- | --- | --- | --- | --- | --- | --- | --- |
|  |  | *r*^2^ | *P* |  | *r*^2^ | *P* |  | *r*^2^ | *P* |  | *r*^2^ | *P* |
| Bacteria | ANPP | **0.729** | **0.040** |  | 0.705 | 0.051 |  | **0.746** | **0.033** |  | **0.740** | **0.036** |
|  | litter | **0.781** | **0.022** |  | **0.755** | **0.030** |  | **0.782** | **0.022** |  | **0.780** | **0.022** |
|  | SR | **0.886** | **0.003** |  | **0.914** | **0.002** |  | 0.622 | 0.100 |  | 0.700 | 0.053 |
|  | Plant *H*' | 0.621 | 0.101 |  | **0.744** | **0.034** |  | 0.421 | 0.299 |  | 0.453 | 0.260 |
|  | SWC | 0.466 | 0.245 |  | 0.639 | 0.088 |  | 0.539 | 0.168 |  | 0.486 | 0.222 |
|  | pH | **-0.836** | **0.010** |  | -0.559 | 0.150 |  | **-0.852** | **0.007** |  | **-0.910** | **0.002** |
|  | SOC | **0.840** | **0.009** |  | 0.686 | 0.060 |  | **0.832** | **0.010** |  | **0.838** | **0.009** |
|  | TN | 0.189 | 0.655 |  | 0.317 | 0.445 |  | 0.399 | 0.327 |  | 0.322 | 0.437 |
|  | C/N ratio | **0.837** | **0.010** |  | 0.679 | 0.064 |  | **0.826** | **0.012** |  | **0.833** | **0.010** |
|  | TP | 0.518 | 0.189 |  | 0.358 | 0.383 |  | 0.409 | 0.315 |  | 0.476 | 0.233 |
|  | NH_4_^+^ | -0.502 | 0.205 |  | -0.615 | 0.105 |  | -0.441 | 0.274 |  | -0.398 | 0.329 |
|  | NO_3_^-^ | **0.909** | **0.002** |  | **0.801** | **0.017** |  | 0.828 | 0.011 |  | **0.885** | **0.003** |
|  | AP | -0.049 | 0.908 |  | -0.263 | 0.530 |  | 0.304 | 0.465 |  | 0.301 | 0.468 |
|  | MC | 0.686 | 0.060 |  | 0.679 | 0.064 |  | 0.695 | 0.056 |  | 0.673 | 0.067 |
|  | MN | 0.656 | 0.077 |  | 0.681 | 0.063 |  | 0.650 | 0.081 |  | 0.628 | 0.096 |
| Fungal | ANPP | -0.093 | 0.826 |  | -0.287 | 0.491 |  | -0.153 | 0.717 |  | -0.041 | 0.924 |
|  | Litter | -0.177 | 0.675 |  | -0.347 | 0.400 |  | -0.254 | 0.544 |  | -0.143 | 0.736 |
|  | SR | -0.239 | 0.569 |  | -0.360 | 0.381 |  | -0.346 | 0.401 |  | -0.256 | 0.540 |
|  | Plant *H*' | -0.365 | 0.374 |  | -0.549 | 0.159 |  | -0.371 | 0.366 |  | -0.323 | 0.435 |
|  | SWC | -0.377 | 0.357 |  | -0.633 | 0.092 |  | -0.365 | 0.374 |  | -0.302 | 0.468 |
|  | pH | 0.151 | 0.722 |  | 0.100 | 0.815 |  | 0.328 | 0.427 |  | 0.174 | 0.680 |
|  | SOC | 0.005 | 0.990 |  | 0.058 | 0.891 |  | -0.072 | 0.866 |  | 0.015 | 0.973 |
|  | TN | -0.501 | 0.206 |  | -0.691 | 0.058 |  | -0.397 | 0.330 |  | -0.345 | 0.403 |
|  | C/N ratio | 0.027 | 0.949 |  | 0.087 | 0.837 |  | -0.052 | 0.902 |  | 0.033 | 0.938 |
|  | TP | -0.302 | 0.467 |  | -0.388 | 0.342 |  | -0.559 | 0.149 |  | -0.459 | 0.253 |
|  | NH_4_^+^ | -0.102 | 0.811 |  | 0.037 | 0.930 |  | -0.029 | 0.946 |  | -0.035 | 0.934 |
|  | NO_3_^-^ | -0.243 | 0.562 |  | -0.305 | 0.462 |  | -0.436 | 0.281 |  | -0.299 | 0.472 |
|  | AP | -0.121 | 0.774 |  | 0.015 | 0.971 |  | -0.029 | 0.945 |  | 0.086 | 0.840 |
|  | MC | -0.202 | 0.632 |  | -0.375 | 0.361 |  | -0.240 | 0.567 |  | -0.155 | 0.714 |
|  | MN | -0.084 | 0.843 |  | -0.350 | 0.395 |  | -0.164 | 0.698 |  | -0.073 | 0.864 |

Bold values represent significant relationships.

Note: ANPP: above-ground net primary productivity; Litter: litter biomass; SR: species richness; plant *H*’: Shannon-Wiener index; OC: soil organic carbon content; TN: soil total nitrogen content; NH_4_^+^: soil NH_4_^+^ content; NO_3_^-:^ soil NO_3_^-^ content; pH: soil pH value; TP: soil total phosphorus content; AP: soil available phosphorus content; SW: soil water content; MC: soil Microbial C biomass; MN: soil Microbial N biomass.

**Figure Captions**

**FIGURE S1** Comparison of main plant species between overgrazing and grazing exclusion. Values represent the mean ± standard error (n = 4). Significance levels are indicated as: ^*^*P* < 0.05, ^**^*P* < 0.01, and ^***^*P* < 0.001.

**FIGURE S2** The rarefaction curves of bacterial (a) and fungal (b).

**FIGURE S3** Comparison of soil bulk density (BD) between overgrazing and grazing exclusion in depth increments of 0–5, 5–10, 10–15, and 10–20 cm. Values represent the mean ± standard error (n = 4). Significance levels are indicated as: ^*^*P* < 0.05, ^**^*P* < 0.01, and ^***^*P* < 0.001.

**FIGURE S4** Proteobacteria class comparison between overgrazing and grazing exclusion. Significance levels are indicated as: ^*^*P* < 0.05, ^**^*P* < 0.01, and ^***^*P* < 0.001.

**FIGURE S5** Comparison of bacterial families with significant differences between overgrazing and grazing exclusion. The data were visualized using STAMP (error bars represent Welch’s t-interval). Bars on the left represent the proportion of each bacterial family’s abundance in the treatments. Bacterial abundance differences with a q-value of < 0.05 were considered to be significant. Q-values are from the adjustment of *p*-values using the Benjamini-Hochberg method.

**FIGURE S6** Comparison of the bacterial genera with significant differences between overgrazing and grazing exclusion. The data were visualized using STAMP (error bars represent Welch’s t-interval). Bars on the left represent the proportion of each bacterial genera’s abundance in the treatments. Bacterial abundance differences with a q-value of < 0.05 were considered to be significant. Q-values are from the adjustment of *p*-values using the Benjamini-Hochberg method.

**FIGURE S7** Comparison of fungal families with significant differences between overgrazing and grazing exclusion. The data were visualized using STAMP (error bars represent Welch’s t-interval). Bars on the left represent the proportion of each fungal family’s abundance in the treatments. Fungal abundance differences with a q-value of < 0.05 were considered to be significant. Q-values are from the adjustment of *p*-values using the Benjamini-Hochberg method.
