# Supplementary figures and images for: Coupling Between the Responses of Plants, Soil, and Microorganisms Following Grazing Exclusion in an Overgrazed Grassland
Source: Front Plant Sci. 2021 Jul 26;12:640789. doi: 10.3389/fpls.2021.640789 (PMC8351616; doi:10.3389/fpls.2021.640789)

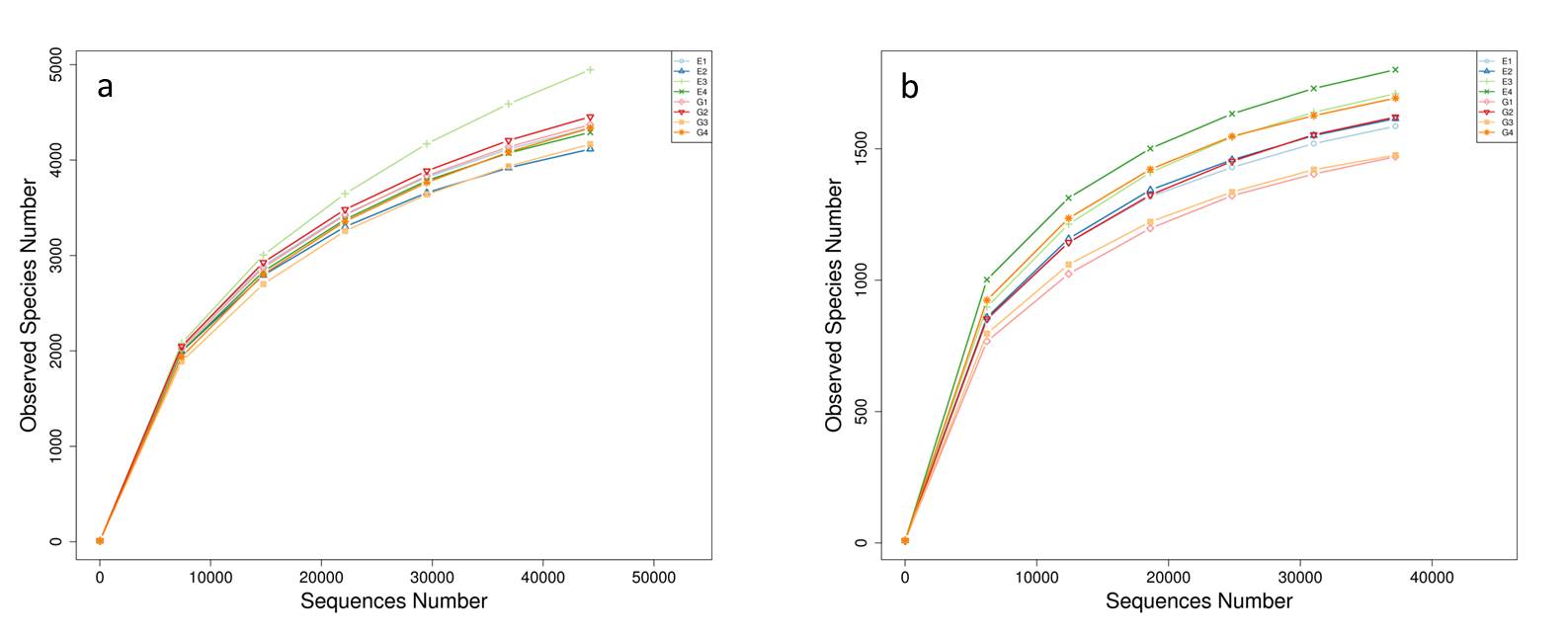

Supplement: Supplementary file 3 [file Image_2.JPEG]

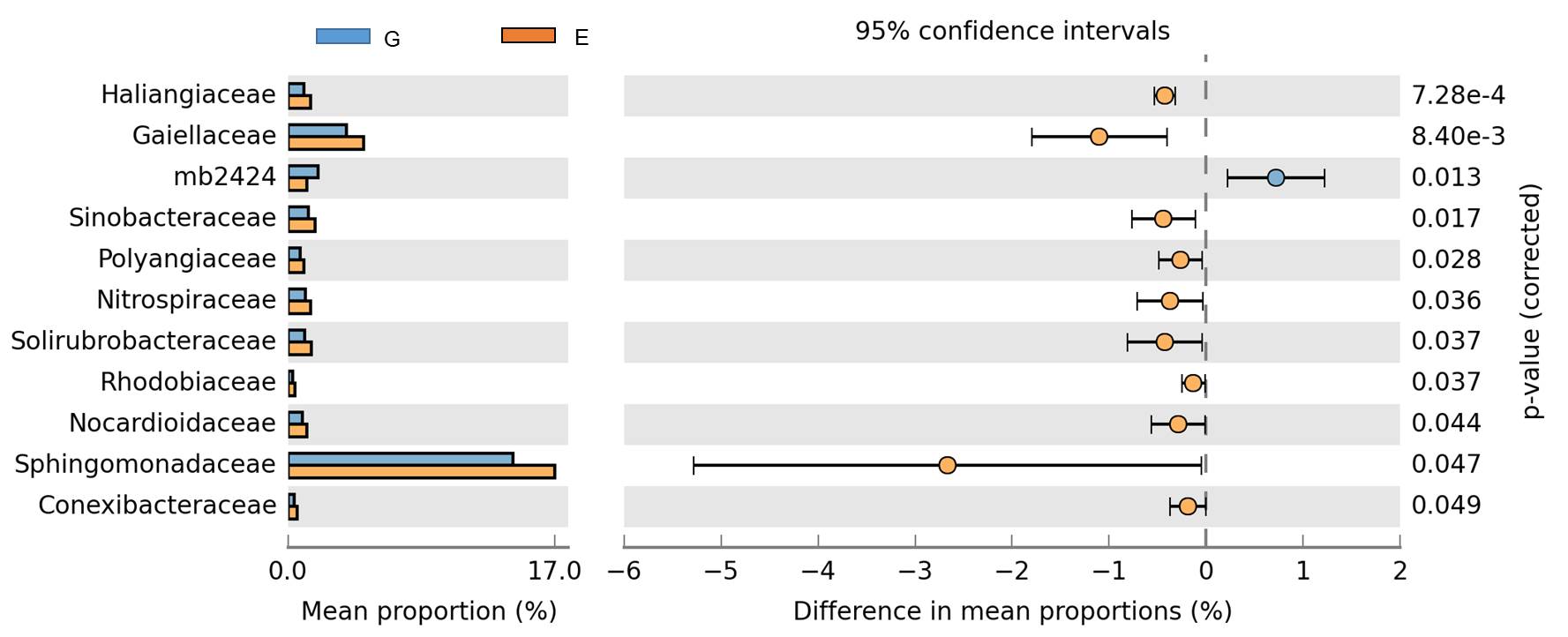

Supplement: Supplementary file 6 [file Image_5.JPEG]

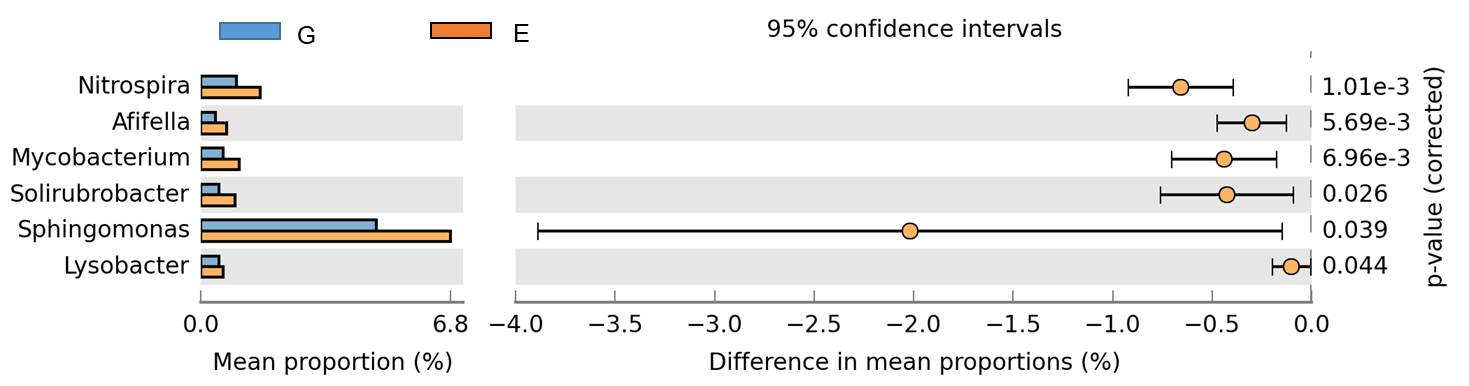

Supplement: Supplementary file 7 [file Image_6.JPEG]

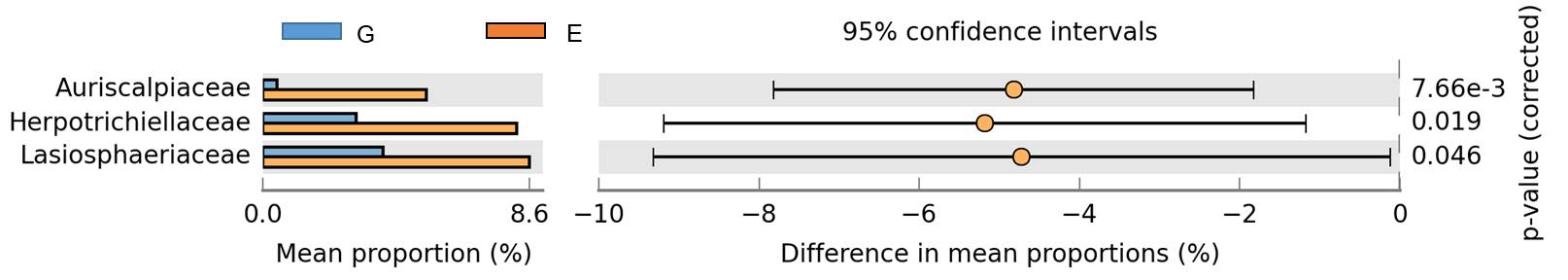

Supplement: Supplementary file 8 [file Image_7.JPEG]
